# The Construct Validity and Reliability of an Assessment Tool for Competency in Cochlear Implant Surgery

**DOI:** 10.1155/2014/192741

**Published:** 2014-07-10

**Authors:** Patorn Piromchai, Pornthep Kasemsiri, Sudanthi Wijewickrema, Ioanna Ioannou, Gregor Kennedy, Stephen O'Leary

**Affiliations:** ^1^Department of Otolaryngology, Royal Victorian Eye and Ear Hospital, University of Melbourne, East Melbourne, VIC 3002, Australia; ^2^Department of Otorhinolaryngology, Faculty of Medicine, Khon Kaen University, Khon Kaen 40002, Thailand; ^3^Centre for the Study of Higher Education, University of Melbourne, Parkville, VIC 3052, Australia

## Abstract

*Introduction*. We introduce a rating tool that objectively evaluates the skills of surgical trainees performing cochlear implant surgery. *Methods*. Seven residents and seven experts performed cochlear implant surgery sessions from mastoidectomy to cochleostomy on a standardized virtual reality temporal bone. A total of twenty-eight assessment videos were recorded and two consultant otolaryngologists evaluated the performance of each participant using these videos. *Results*. Interrater reliability was calculated using the intraclass correlation coefficient for both the global and checklist components of the assessment instrument. The overall agreement was high. The construct validity of this instrument was strongly supported by the significantly higher scores in the expert group for both components. *Conclusion*. Our results indicate that the proposed assessment tool for cochlear implant surgery is reliable, accurate, and easy to use. This instrument can thus be used to provide objective feedback on overall and task-specific competency in cochlear implantation.

## 1. Introduction

Training in surgery for cochlear implantation is generally undertaken at the postfellowship level, by surgeons joining or starting a cochlear implant program. As such, surgeons learning this operation need to schedule training time around busy clinical commitments, so efficiency of learning is a key requirement. In addition, surgeons beginning cochlear implantation (CI) will already have received foundational otological training, so the focus of training should be upon nuances relating to the implant procedure. These specific requirements for CI training should be considerations when evaluating a new implant surgeon's competence and readiness for independent practice.

Log-book based assessments, based upon traditional mentorship and accreditation models, are not well suited to this task, given that case-loads for CI surgery are low and opportunities for supervised surgical experience may be limited. Objective assessment of the technical skills required for CI holds greater promise, given that this approach can provide reliable and valid outcomes [1–3], and gives the surgical trainees feedback on specific aspects of their surgical technique that may require further attention. An objective assessment method has the advantage that it could be applied to training in the operating theatre, cadaveric dissection, or simulated surgical training. This means that assessment could be provided to surgeons attending intensive CI training courses. The possibility of video recording the surgical exercise also opens up the option that an assessor could rate and provide feedback at a later time, freeing the surgeons to schedule training exercises independently around their practice commitments.

Only a few instruments have been developed to date for the assessment of objective technical skills in mastoid surgery. Zirkle et al. [4] and Laeeq et al. [5] developed tools for the evaluation of competency in mastoidectomy based on the objective structured assessment of technical skills (OSATS) approach. An OSATS uses three scoring systems for each task station: a task-specific checklist, a global rating scale, and a pass/fail judgment by the assessor on the performance. This approach has been found to be a reliable and valid method to assess surgical skills [1–3]. Butler and Wiet [6] introduced an “end-product” assessment tool for mastoidectomy, namely, the “Welling scale,” that used a 35-item binary (0, 1) grading instrument of a completed cortical mastoidectomy and validated it with PGY-3 residents. Zhao et al. extended the Welling scale, introducing global rating and indices of drilling technique in their assessment of residents trained in a virtual environment [7, 8]. More recently, Wan and colleagues proposed a Cross-Institutional Temporal Bone Dissection Grading Scale based on similar principles to those of Welling's, after garnering the expert opinion of American otologists by questionnaire [9].

To our knowledge, there does not exist an objective assessment tool for cochlear implant surgery. Assessment tools for this procedure will differ from those developed for temporal bone dissection because the latter is aimed at the general otolaryngologist, whereas cochlear implantation training targets a specific operation performed by otologic subspecialists. A majority of training institutes do not include cochlear implant training as a routine part of residency training. This is supported by the observation that cochlear implantation is beyond the abilities of a resident in the program director survey [10]. Here we introduce a two-component rating tool comprising of (1) a global rating score to rate the overall surgical skills relevant to this surgical procedure and (2) a task-based checklist to evaluate competence in preparing the surgical approach for cochlear implantation. The tool was evaluated in a virtual temporal bone environment, and its sensitivity was assessed by comparing ratings amongst resident and expert cochlear implant surgeons.

## 2. Materials and Methods

### 2.1. Assessment Instrument

The cochlear implant surgery performance rating tool was developed by three consultant otolaryngologists and one medical education expert. The content of each item was arrived at by consensus. This assessment tool's design was based on the OSATS since it has been commonly used in the medical educational community for more than a decade. We reasoned that the familiarity of the OSATS structure would increase the usability of the assessment tool.

The proposed tool comprises two components: a global competency scale (Table 4) and a task-based checklist (Table 5). The global competency scale is based on the work of Reznick [11] and Laeeq et al. [5] and consists of seven items that assess preparation and processes. The task-based checklist is based on the seven procedural steps in our course curriculum and standard textbooks [12]. Items in both sections of the cochlear implant surgery assessment tool are scored on a five-point Likert scale, with descriptors at midpoint and extremes [13].

### 2.2. Ethical Considerations

Ethical approval was provided by the Royal Victorian Eye and Ear Hospital Ethics Committee. All subjects were otolaryngology trainees and consultants who volunteered to perform cortical mastoidectomy and cochleostomy via a facial recess approach on a virtual reality temporal bone and provide information for this trial under the guidelines of the approved protocol. They were provided with surgical instruction tailored to optimize the procedure for cochlear implantation.

### 2.3. Experimental Procedure

The residents and consultants were given standard instruction to perform this surgical approach to cochlear implantation on a standardized virtual reality temporal bone. None of the participants had prior experience in using the simulator. All participants were introduced to the virtual environment and given ten to fifteen minutes to familiarize themselves with the virtual environment; they were asked to perform the surgery as they would on a real patient. The participants were asked to perform at least 2 surgical sessions.

The virtual reality system was based on the University of Melbourne temporal bone simulator that was validated for its face and content validity [7, 8]. The system was composed of two Intel Xeon W3565 processors at 3.2 GHz with 12 GB main memory, an nVIDIA Quadro 4000 graphics card, and a 24-inch liquid crystal display (LCD) monitor with in-plane switching (IPS) technology. Two slightly offset images are projected onto the screen, and when viewed through a pair of nVIDIA 3D vision shutter glasses, a three-dimensional illusion of the operating space is observed. The user interacts with the virtual reality system by using a PHANTOM Desktop haptic device. The haptic device is represented in the system as a virtual drill and haptic feedback is provided to the user when the drill interacts with the operating space.

The temporal bone data was derived from microcomputed tomography with a voxel resolution of 96 × 96 × 96 *μ*m and cropped into a 155 × 230 × 255 volume. The anatomical structures were segmented manually and then rendered in 3D. The specific anatomical features presented were the facial nerve, chorda tympani, malleus, incus, stapes, cochlea, semicircular canals, dura mater, stapedius tendon, basilar membrane, round window membrane, and sigmoid sinus.

Each procedure was recorded separately as a continuous data stream on the virtual reality system and using video capture software. To minimize the bias, we did not isolate segments of the video or shorten the length of each procedural stage. The evaluator needed to watch the video from the start to the end. The participants were identified by a code and no participant information was shown in either the recordings on the virtual reality system or the videos. The videos were sent in a random sequence and reviewed independently by two consultant otolaryngologists who were not made aware of any participant information including their identity. They scored the performance using the global rating scale and the task-based checklist.

### 2.4. Statistical Analysis

Statistical analysis was performed using the Statistical Package for the Social Sciences (SPSS) version 20.0. Data was described by either means for continuous variables or frequencies and percentages for categorical variables.

To assess reliability, we derived the intraclass correlation coefficient with a 2-way random model for interrater agreement. We report 95% confidence intervals (CI), which provide information on the precision of correlation of rater agreement. In addition, we record *P* values and consider the results to be statistically significant if *P* < 0.05.

## 3. Results

Seven residents and seven experts participated in the cochlear implant surgery sessions. A total of 28 assessment videos were recorded, with 16 having been performed by consultants (5 consultants performed 2 sessions, and 2 consultants performed 3 sessions) and 12 by residents (5 residents performed 2 sessions, and 2 residents performed 1 session). Two evaluators assessed all these videos and provided feedback on the assessment tool.

Interrater reliability was calculated as intraclass correlation coefficient (ICC) for both global and checklist components of the assessment instrument (Tables 1 and 2). For the global assessment scale, overall ICC was scored as high (ICC 0.96, 95% CI 0.93 to 0.98, *P* < 0.001). The highest agreement (ICC 0.86, 95% CI 0.70 to 0.94, *P* < 0.001) was recorded for “knowledge of specific procedure” and the lowest agreement (ICC 0.67, 95% CI 0.28 to 0.85, *P* = 0.003) was for “use of otologic drill.” For the checklist component of the rating tool, the overall ICC was also observed to be high (ICC 0.92, 95% CI 0.86 to 0.96, *P* < 0.001). The highest agreement (ICC 0.93, 95% CI 0.84 to 0.97, *P* < 0.001) was observed for “facial recess is opened to visualize the round window niche” and the lowest agreement (ICC 0.69, 95% CI 0.31 to 0.86, *P* = 0.003) was for “thin posterior EAC cortex maximally.”

The construct validity of this instrument is strongly supported by the significantly higher overall score of the expert group when compared to that of residents (see Table 3). The mean difference in the global rating score for the two groups was 8.14 (95% CI 4.36 to 11.91, *P* < 0.001) and for the task-based checklist it was 8.24 (95% CI 4.10 to 12.38, *P* < 0.001).

**Figure 1:**
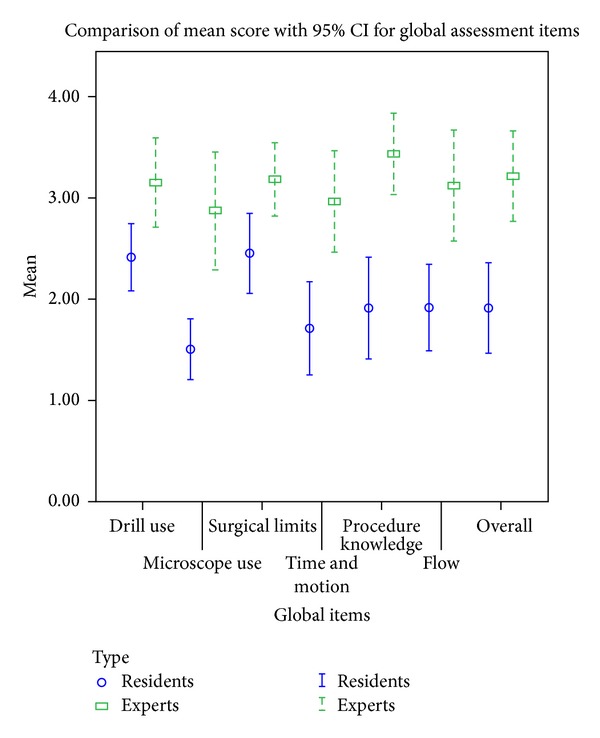
Comparison of mean score with 95% CI for global assessment items.

Each individual item of the two assessment components also demonstrated a high level of construct validity. The difference in performance among experts and residents was observed to be more prominent when the participants performed more complex steps related to facial recess and cochleostomy. For the global scale, all items were significantly different between experts and residents (*P* < 0.05). The items where the performance of the two groups differed to the greatest extent were “knowledge of specific procedure” (mean difference 1.52, 95% CI 0.92 to 2.12, *P* < 0.001) and “use of microscope” (mean difference 1.37, 95% CI 0.68 to 2.07, *P* < 0.001) (Figure 1).

**Figure 2:**
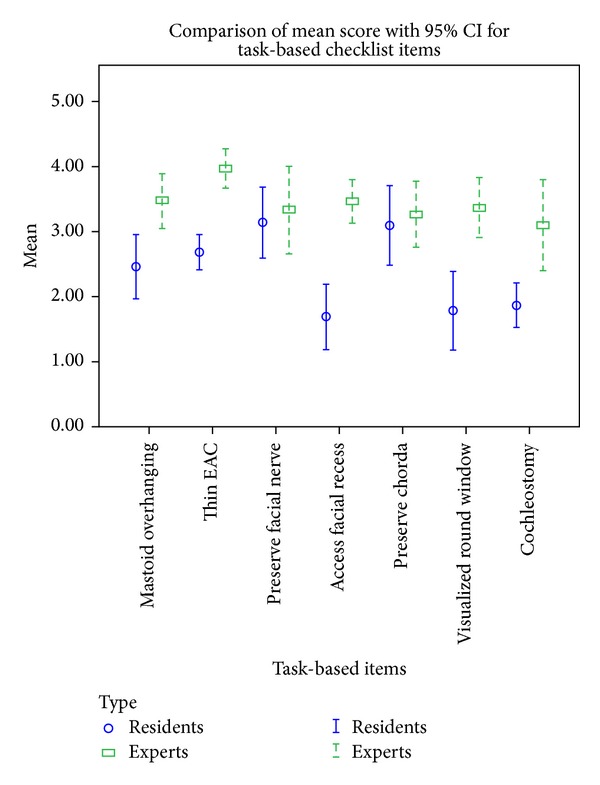
Comparison of mean score with 95% CI for task-based checklist items.

For the task-based checklist, 5 out of 7 items were significantly different in the two groups (*P* < 0.05). The items that showed the highest difference between the expert and resident groups were “remove bone on the anterior medial surface of the fallopian canal just below the pyramidal process” (mean difference 1.72, 95% CI 1.15 to 2.30, *P* < 0.001) and “facial recess is opened to visualize the round window niche” (mean difference 1.51, 95% CI 0.78 to 2.24, *P* < 0.001). The least significant items were “preserve a layer of bone overlying the facial nerve” and “preserve a layer of bone overlying the chorda tympani” (Figure 2).

## 4. Discussion

Objective assessment of technical skills to demonstrate surgical competency in the field of otolaryngology has been receiving increasing attention in recent years [10]. However, valid and reliable evaluation of real-life skills in the operating room is still in its infancy, specifically with regards to cochlear implant surgery. In this paper, we introduced a two-component assessment tool in an attempt to bridge this gap. The first component was a global rating score to assess the overall surgical competency and the second was a task-based checklist to evaluate the performance in the cochlear implantation. The tool performed reasonably well, with high levels of interrater agreement and sufficient sensitivity to differentiate between surgical residents and experienced cochlear implant surgeons. The fact that there were significant differences in the scores obtained from most of the individual items on the global and task-specific scales suggests that the tool can provide feedback on specific aspects of technique, helping the surgeons to identify strengths in their technique and areas requiring further improvement.

### 4.1. Global Rating Scale

The indices of the global rating scale of this study were derived from the objective structured assessment of technical skill (OSATS) for surgical residents that was created by Martin et al. [2] in 1997. These authors proposed a global assessment tool based on three factors fundamental to surgical technique, namely, complex visuospatial organization, stress tolerance, and psychomotor abilities [14, 15]. Martin found that the OSATS global rating scale could discriminate between resident groups with differing levels of experience. Global rating scales have subsequently been incorporated into validated assessment tools for competency in mastoidectomy [5] that have been designed for use in resident training. Zirkle et al. [4] also used the global rating scale in their objective assessment of temporal bone drilling skills for otolaryngology residents in Toronto, Canada. Their scale assessed stroke, grip, direction, and chatter of the surgical drill.

The global assessment tool in this study aimed to assess the overall readiness of the trainee to undertake cochlear implant surgery. To evaluate the trainees' psychomotor skills and knowledge of the procedure, seven items were considered. The first of these was use of the otologic drill. Good surgical technique is reflected in the appropriate choice of burr and drilling with long and smooth strokes that run parallel to underlying structures [16]. Failure to demonstrate competency here suggests that the trainee has not acquired the drill-handling skills required to prepare the temporal bone for cochlear implantation. The second item was the use of the operating microscope. The later stages of mastoidectomy should always be done under the microscope [16] with appropriate patient positioning and magnification. Skill with the microscope will be more important when the trainees approach the facial recess, in order to achieve the best visualization of the round window and to facilitate the cochleostomy [17]. The third item is tissue handling; trainees need to carefully handle tissues with proper technique and know when to abort to prevent further damage. Ignorance of these surgical limits may lead to both major and minor complications, for example, facial nerve injury [18]. The fourth item is time and motion. The trainees should perform the operation in a timely manner. The average surgical time for cochlear implant surgery in the academic setting has been reported as 2.5 to 3 hours [19], and for this reason the trainees were expected to complete the cochleostomy in less than 2 hours. The next two items were knowledge of the specific procedure and the flow of the operation. Disruptions of surgical flow have been associated with increased rates of surgical error [20], so it is important that trainees demonstrate a smooth progression through the steps of the operation. A good understanding of the operation clearly underpins this, as do the handling of the drill and the familiarity with the surgical environment, and therefore flow is a good indicator of the trainee's synthesis of these technical factors.

It may seem surprising that the expert scores using the global rating scale were relatively low across all items. This is particularly the case for the item “knowledge of specific procedure” because the experts would have been expected to have scored perfect scores. The less-than-perfect scores may be related to these experts performing the operation in the virtual reality system, an environment with which they were not familiar. This phenomenon also appears in a previous work on temporal bone dissection in virtual reality [4], where Zirkle noted that experts achieved a mean global rating score of 10 out of 16. These discrepancies might also relate to the method of assessment, given the reliance upon video recordings which were analysed blinded, without the opportunity to interrogate the participants and clarify their knowledge.

### 4.2. Task-Based Checklist

The task-based checklist of this study was also based on OSATS [2, 15, 21]. Laeeq et al. [5] developed a task-based checklist for mastoidectomy. Items in this checklist were grouped by their contribution to procedural goals of increasing complexity. These authors combined both a task-list and a global rating scale and found that the tool was reliable and valid in both sections. Zirkle et al. [4] created the task-based checklist consisting of 16 binary items (0/1) and combined this with a global rating scale and a final product analysis scale of the drilled temporal bone.

The cochlear implant surgery global rating tool presented here was based on those developed for mastoidectomy and temporal bone dissection, together with specific technical aspects that were identified in the literature. To our knowledge, there has been no prior study to validate a task-based checklist for this procedure. The basis for the competency rating tool was first drafted based on the steps outlined in a standard text [12] and revised through consideration of the literature and consensus between the investigators that brought perspectives in surgery and educational psychology from a major western cochlear implant clinic and a large Asian university.

The first of the seven items selected was “left slightly overhanging superiorly, posteriorly, and inferiorly to help retain and control the electrode cable.” Mastoidectomy is the important first step in the cochlear implant surgery to adequately visualize the facial recess and middle ear. It is nuanced in cochlear implantation by leaving a bony overhang at the margins of the mastoid that is helpful in retaining the electrode array, which is coiled into the mastoid cavity [22]. This is thought to reduce the risk of electrode migration, which is of particular significance not only for cochlear implantation in children 6 to 12 months of age [17], but also for straight electrodes that are not held within the cochlea [22].

The next item included was “thin the posterior EAC cortex maximally.” If the posterior external auditory canal is not thinned adequately, the angle through the facial recess becomes more difficult to negotiate and the round window niche cannot often be visualized. If the round window niche is not seen, then it is not possible to place the cochlear electrode accurately [17, 23]. On the other hand, the posterior EAC should not be perforated, as the electrode may extrude through this iatrogenic hole into the ear canal.

Also included was “preserve a layer of bone overlying the facial nerve.” While identification of the facial nerve is the key to performing safe cochlear implant surgery, facial nerve palsy is a risk and one of the most serious potential complications. Injury to the facial nerve may occur due to mechanical trauma or thermal injury from inadequate irrigation whilst using the drill or applying overly aggressive pressure [24, 25]. Preservation of a layer of bone over the nerve is standard practice in order to minimize the risk of direct trauma from the drill [24].

The fourth item was “remove bone on the anterior medial surface of the fallopian canal just below the pyramidal process.” To facilitate visualization of the round window niche which is the primary landmark for cochleostomy or round window electrode insertion, it is usually necessary to remove bone on the anterior medial surface of the fallopian canal just below the pyramidal process [12, 26–28] because the round window niche is located posteriorly in the mesotympanum. It is good practice therefore to skeletonize both the medial and lateral sides of the facial nerve in order to facilitate visualization of the promontory.

Similarly, we considered “preservation of a layer of bone overlying the chorda tympani.” The permanent chorda tympani syndrome is one of the three most common complications following cochlear implant surgery [29]. Preservation of the bone overlying the chorda tympani may prevent this complication, and yet skeletonization of the structure is required to optimize visualization of the round window [28]. In patients with a narrow facial recess, some surgeons may suspend, place anteriorly, or adhere the chorda tympani nerve to the posterior wall of the auditory canal in order to expose the round window and preserve the function of chorda tympani nerve [30].

The sixth item was that the “facial recess is opened to visualize the round window niche.” In addition to careful dissection of facial nerve and the chorda tympani, the facial recess shape must be optimized to ensure full visualization of the round window and the basal cochlear turn [17, 28, 31]. This is necessary not only to ensure that the surgical landmarks for electrode insertion are identified, but also to facilitate the optimal orientation of the electrode during insertion [28, 31], which can be restricted if the recess is not opened sufficiently, particularly at its superior aspect [31].

The final item was the creation of the cochleostomy or preparation of the round window for electrode insertion. A cochleostomy is drilled through the promontory just anterior and inferior to the round window membrane. Correct placement of the cochleostomy is essential in order to avoid injury to the spiral ligament and to minimize the risk of inadvertent electrode insertion into the scalae media or vestibuli [23, 28, 31, 32]. Despite the prominence of this matter in the literature, a recent survey of practicing otologists found that a surprisingly high proportion place their cochleostomy in too anterior or superior a position to avoid injury to intracochlear structures [23]. Correct orientation of a cochleostomy relative to the round window requires that this structure is visualized fully [23], and the latter necessitates removal of the overhanging niche [12]. For the round window approach, full visualization of the round window must also be achieved, and this too requires removal of the bony overhang on the superior aspect of the round window niche [31] and at times the anteroinferior bony lip (crista semilunaris). These steps are required so that the electrode can be inserted in a posterosuperior to anteroinferior orientation, tangential to the round window in order to achieve a trajectory parallel to the basal cochlear turn that avoids injury to the medial cochlear wall or basilar membrane [31, 33].

For the task-based checklist, 5 of 7 items were significantly different in the two groups (*P* < 0.05). The items where a significant difference was not found were “preservation of a layer of bone overlying the facial nerve” and “preservation of a layer of bone overlying the chorda tympani.” The reason(s) why scores did not differ significantly between the groups for these items is difficult to ascertain from the present study. One potential explanation for this is that resident staff spent more time and care on these steps of the operation, given the importance of not damaging the facial nerve or the chorda. Another possibility is that, in the simulated environment, it may have been more difficult to maintain a bony layer over the facial nerve and chorda tympani due to the properties of the bone removal and bone rendering algorithms. This could potentially explain why experts did not score as highly as might otherwise have been expected. Further insights into why experts did not do better in these test items may be gained by exploring their performance on the OSATS during cadaveric dissection, where it might be expected that expert surgeons would achieve better scores on these test items.

### 4.3. Benefit of the Objective Assessment Tool for Cochlear Implant Surgery

The format of the OSATS, with both global and task-based rating scales, will assist trainers in providing feedback to surgeons on their readiness to undertake training for CI surgery. A surgeon who does not reach an acceptable standard on the global rating scale may be advised to gain further otological experience prior to considering taking up CI surgery. An experienced otologist with a good global rating scale should find the feedback on aspects of the surgery needing further attention helpful for improving their skills. This level of detail may also assist educators evaluate their own training programs; strengths and weaknesses of a particular program could potentially be inferred from participants' performance on the task-based scale.

The assessment tools that have been developed here could potentially be used to evaluate surgical performance during cadaveric dissection, or in the operating theatre. We predict that the difference between experts and trainees may be even greater in these situations, given that experienced surgeons will find these environments more familiar than the virtual simulated one.

## 5. Conclusion(s)

Our results indicate that the proposed assessment tool is a valid and reliable method of evaluating the technical skills for cochlear implant surgery. It can be used to provide objective feedback on overall and task-specific competency in the surgical approach to cochlear implantation.

## Figures and Tables

**Table 1:** Interrater agreement for global assessment scale.

Items	Intraclass correlation coefficient (ICC)	95% CI	*P* value
Use of otologic drill	0.67	0.28 to 0.85	0.003
Use of microscope	0.79	0.54 to 0.90	<0.001
Respect for surgical limits	0.79	0.55 to 0.90	<0.001
Time and motion	0.79	0.47 to 0.91	<0.001
Knowledge of specific procedure	0.86	0.70 to 0.94	<0.001
Flow of operation	0.82	0.57 to 0.92	<0.001
Overall surgical performance	0.80	0.56 to 0.91	<0.001

All variables	0.96	0.93 to 0.98	<0.001

**Table 2:** Interrater agreement for task-based checklist.

Items	Intraclass correlation coefficient (ICC)	95% CI	*P* value
Left slightly overhanging superiorly, posteriorly, and inferiorly to help retain and control the electrode cable	0.86	0.66 to 0.94	<0.001
Thin posterior EAC cortex maximally	0.69	0.31 to 0.86	0.003
Preserve a layer of bone overlying the facial nerve	0.85	0.68 to 0.93	<0.001
Remove bone on the anterior medial surface of the fallopian canal just below the pyramidal process	0.74	0.44 to 0.88	<0.001
Preserve a layer of bone overlying the chorda tympani	0.84	0.66 to 0.93	<0.001
Facial recess is opened to visualize the round window niche	0.93	0.84 to 0.97	<0.001
Cochleostomy is drilled through the promontory just anterior and inferior to the round window membrane	0.79	0.53 to 0.91	0.037
The bony promontory overlying the round window niche has been removed	—		—

All variables	0.92	0.86 to 0.96	<0.001

**Table 3:** Mean competency scores between experts and residents.

Parts	Experts (*n* = 16)	Residents (*n* = 12)	Mean difference	95% CI	*P* value
Global rating score	21.97	13.83	8.14	4.36 to 11.91	<0.001
Task-based checklist score	23.53	15.29	8.24	4.10 to 12.38	<0.001

**Table 4:** The global rating scale for cochlear implantation competency.

(1) Use of otologic drill	1	2	3	4	5
	Chooses inappropriate burr and/or repeatedly awkward use of drill		Chooses appropriate burr and occasionally awkward use of drill		Uses appropriate burr and drill effortlessly

(2) Use of microscope	1	2	3	4	5

	Repeatedly inappropriate position, magnification, or focus		Competent use of microscope but occasional inappropriate position and magnification		Optimal visualization with appropriate microscope use

(3) Respect for surgical limits	1	2	3	4	5

	Uses unnecessary force or causes damage by inappropriate use of instruments		Careful handling of tissue but occasional inadvertent damage to tissue		Consistently handled tissues appropriately with minimal damage

(4) Time and motion	1	2	3	4	5

	Many unnecessary movements		Efficient time/motion and maximum efficiency		Clear economy of movement

(5) Knowledge of specific procedure	1	2	3	4	5

	Deficient knowledge and needed instruction at most steps		Knew all important steps of operation		Demonstrated familiarity with all aspects of operation

(6) Flow of operation	1	2	3	4	5

	Frequently stopped and unsure of next move		Some forward planning with reasonable progression		Obviously planned course of operation with effortless flow

(7) Overall surgical performance	1	2	3	4	5

	Poor		Performs majority of surgery acceptably		Outstanding

**Table 5:** The task-based checklist for cochlear implantation competency.

Tasks	Poor		Acceptable		Outstanding	
*Mastoidectomy *						
(1) Left slightly overhanging superiorly, posteriorly, and inferiorly to help retain and control the electrode cable	1	2	3	4	5	N/A
(2) Thin posterior EAC cortex maximally	1	2	3	4	5	N/A

*Facial recess opening *						
(3) Preserve a layer of bone overlying the facial nerve	1	2	3	4	5	N/A
(4) Remove bone on the anterior medial surface of the fallopian canal just below the pyramidal process	1	2	3	4	5	N/A
(5) Preserve a layer of bone overlying the chorda tympani	1	2	3	4	5	N/A
(6) Facial recess is opened to visualize the round window niche	1	2	3	4	5	N/A

*Cochleostomy *						
Conventional approach						
(7) Cochleostomy is drilled through the promontory just anterior and inferior to the round window membrane	1	2	3	4	5	N/A
Round window approach						
(8) The bony promontory overlying the round window niche has been removed	1	2	3	4	5	N/A
